# Pharmacologic and Acute Management of Spinal Cord Injury in Adults and Children

**DOI:** 10.1007/s11940-022-00720-9

**Published:** 2022-06-10

**Authors:** Ajay X. Thomas, James J. Riviello, Daniel Davila-Williams, Sruthi P. Thomas, Jennifer C. Erklauer, David F. Bauer, Jon A. Cokley

**Affiliations:** 1 Division of Pediatric Neurology and Developmental Neuroscience, Department of Pediatrics, Baylor College of Medicine, Houston, TX USA; 2 Jan and Dan Duncan Neurological Research Institute, Texas Children’s Hospital, Houston, TX USA; 3 Division of Pediatric Physical Medicine and Rehabilitation, Department of Physical Medicine and Rehabilitation, Baylor College of Medicine, Houston, TX USA; 4 Division of Pediatric Neurosurgery, Department of Neurosurgery, Baylor College of Medicine, Houston, TX USA; 5 Division of Pediatric Critical Care Medicine, Department of Pediatrics, Baylor College of Medicine, Houston, TX USA; 6 Department of Pharmacy, Baylor College of Medicine, Houston, TX USA

**Keywords:** Spinal cord injury, Surgical management of vertebral fracture, MAP management, Spinal shock, Autonomic dysreflexia, Rehabilitation

## Abstract

**Purpose of Review:**

This review provides guidance for acute spinal cord injury (SCI) management through an analytical assessment of the most recent evidence on therapies available for treating SCI, including newer therapies under investigation. We present an approach to the SCI patient starting at presentation to acute rehabilitation and prognostication, with additional emphasis on the pediatric population when evidence is available.

**Recent Findings:**

Further studies since the Surgical Timing in Acute Spinal Cord Injury Study (STASCIS) demonstrated a potential functional outcome benefit with ultra-early surgical intervention ≤ 8 h post-SCI. Subsequent analysis of the National Acute Spinal Cord Injury Study (NASCIS) II and NASCIS III trials have demonstrated potentially serious complications from intravenous methylprednisolone with limited benefit. Newer therapies actively being studied have demonstrated limited or no benefit in preclinical and clinical trials with insufficient evidence to support use in acute SCI treatment.

**Summary:**

Care for SCI patients requires a multi-disciplinary team. Immediate evaluation and management are focused on preventing additional injury and restoring perfusion to the affected cord. Rapid assessment and intervention involve focused neurological examination, targeted imaging, and surgical intervention when indicated. There are currently no evidence-based recommendations for pathomechanistically targeted therapies.

## Introduction

Annually, there are approximately 54 new spinal cord injuries (SCI) per one million people in the USA, nearly 17,900 new cases per year based on 2020 estimates. Roughly 296,000 people live with SCI in the USA [1]. The morbidity associated with SCI has a substantial impact on the healthcare system. Lifetime care of traumatic SCI (tSCI) patients ranges from $1,600,000 to $4,800,000 [1]. Given the staggering impact of SCI on society, proper care of these patients is paramount. Immediate evaluation and management are focused on preventing further injury to the spinal cord, which can be caused by spine instability, spinal cord compression, systemic hypotension, vascular compromise, and elevated intrathecal pressure.

## Initial Evaluation

### Examination

Early recognition is imperative to avoid additional damage, especially during transport. Patients are at risk for airway compromise, respiratory failure, and circulatory failure. Initial evaluation begins with assessing the airway, breathing, and circulation (ABCs) [2••]. Other components of the examination include the patient’s Glasgow Coma Scale, pupil size and reactivity, extremity movement, and SCI level based on motor and sensory deficits. Additional signs should be evaluated, including loss of rectal tone, hyperreflexia/areflexia, and sacral exam (anal reflex and/or bulbocavernosus reflex) [2••, 3••]. Typically, reflex arcs at or below the level of the injury are impaired; however, more distal reflexes such as the anal and bulbocavernosus reflex may remain intact [4].

### Imaging

Patients presenting with suspected acute tSCI require imaging to evaluate the extent of osseus and spinal cord parenchymal injury, to evaluate for ongoing mechanisms of injury or compression, and to determine spinal stability. Extent of injury provides prognosis while ongoing compression or instability informs treatment. Initial spinal computed tomography (CT) scan should be rapidly obtained to evaluate for fracture. In patients with neurologic deficit, emergent magnetic resonance imaging (MRI) is indicated to evaluate for disc herniation, epidural hematoma, spinal cord contusion or injury, and ligamentous injury. CT angiogram can evaluate for vertebral artery injury in the cervical spine or aorto-iliac injury in the thoracolumbar spine. In the subacute setting, dynamic flexion and extension imaging can evaluate for instability. Historically, dynamic imaging consisted of radiographs; however, flexion extension CT and MRI are now often utilized [5].

Validated clinical radiographic scoring systems may predict the need for operative fixation and arthrodesis, such as the modern Subaxial Cervical Spine Injury Classification (SLIC), Thoracolumbar Injury Classification and Severity Scale (TLICS), and AOSpine, including the pioneering White and Panjabi score. Historically, fracture types were used to predict stability. The White and Panjabi model of the spine based on three structural columns (anterior vertebral body, posterior vertebral body, and facet complex) posited when two or three columns were injured, the fracture was unstable [6, 7]. The modern classification systems use CT, MRI, and clinical data to better predict fracture stability and need for surgery. The SLIC system gives points for fracture morphology, integrity of disc-ligamentous complex, and patient neurologic status on a scale from 0 to 10; non-operative treatment is recommended for scores < 4, and operative treatment is recommended for scores ≥ 5 [8]. Since inter-rater reliability can be variable, new classifications systems continue to be published.

Non-traumatic SCI presenting acutely may necessitate a similar work-up; however, early MRI may be indicated to detect spinal tumor, epidural abscess, spontaneous spinal cord hemorrhage, spine syrinx, acute demyelination, or other non-traumatic etiologies. Conventional angiogram or CT angiogram may be indicated to evaluate for arteriovenous malformation (AVM) or arteriovenous fistula. A minor trauma with neurologic deficit out of proportion to the injury may suggest one of these etiologies but can be associated with Chiari malformation as well. Pediatric patients are also susceptible to spinal cord injury without radiologic abnormality (SCIWORA), which can be seen in up to a quarter of pediatric patients presenting with SCI; therefore, careful evaluation is critical [9]. Notably, the radiological modality classically refers to CT imaging.

## Etiology

There are several distinct causes of SCI. Most share clinical characteristics; however, a thorough evaluation and better understanding of etiology are required to guide possible further therapies, prevent complications, and define prognosis for recovery.

### Trauma

TSCI represents a significant cause of neurological morbidity in the USA [1, 10]. The most common cause of tSCI is motor vehicle accidents, accounting for more than one third of adult cases, followed by falls, the most common cause in elderly patients. Violence and sports-related injuries are also common, especially in children [1, 10, 11]. Most injuries related to trauma affect the cervical cord. The predominant mechanism of injury is direct impact due to mechanical forces associated with cord compression, fracture/displacement of the vertebrae, and/or cord laceration/transection. Secondary mechanisms of injury include hypoxia, hypoperfusion, inflammation, swelling, microhemorrhages, and thrombosis among others [12–15].

### Vascular

Although rare, vascular causes of SCI include ischemic and hemorrhagic infarctions. The most common cause of ischemic injury is related to surgical procedures involving the thoracic or abdominal aorta [16]. Risk factors include peri-procedural systemic hypotension, aortic cross-clamping, and increased spinal canal pressure [17]. Other surgical procedures have also been associated with spinal cord infarction.

Other causes include stenosis or occlusion of the arteries supplying the spinal cord (anterior spinal artery, artery of Adamkiewicz (thoracic spine), and posterior spinal arteries). Aortic dissection can also cause infarction, especially in adults. Fibrocartilaginous embolism (FCE) is a rare cause of spinal cord infarction. It is thought that FCE results from embolized fibrocartilaginous material from intervertebral discs into the spinal vascular system after minor head/neck injury causing occlusion and ischemia [18].

Vascular malformations can also cause SCI. The most common types include dural arteriovenous fistula and intramedullary spinal AVM. Injury is typically secondary to the mass effect of the malformation or by ischemia/hemorrhage into the cord. The clinical presentation could be progressive rather than sudden or acute.

Spinal epidural hematoma is typically seen in tSCI but has also been documented in patients with coagulopathy or those receiving anti-thrombotic therapy [12, 19]. The main clinical features include acute severe back pain followed by weakness/paralysis.

### Compression

Compressive SCI can be seen secondary to benign and malignant tumors. Injury commonly results from external compression, caused by metastases to the extradural space or intramedullary growth. Clinical presentation is typically progressive weakness below the level of the lesion, associated with sensory symptoms and/or bladder dysfunction, and aching back pain (which may occur prior to neurological symptoms). Compression can occur in the thoracic (60%), lumbar (25%), and cervical spine (15%) [12].

### Inflammatory/Infectious

A bacterial infection, such as epidural abscess, is rare but constitutes a neurosurgical emergency. Myelitis occurs with bacterial meningitis, especially streptococcal meningitis. A virus may selectively involve anterior horn cells, such as poliovirus, enterovirus (E71, D68), and West Nile virus. Recent outbreaks of acute flaccid myelitis (AFM) causing a polio-like presentation occur with enterovirus D68. Polio causes asymmetric lower extremity weakness, whereas AFM usually involves the upper extremities.

Acute transverse myelitis/myelopathy (ATM) is an immune-mediated disorder. Inflammatory conditions, such as neuromyeltis optica (aquaporin-4) or myelin oligodendrocyte antibody associated disease (MOG), have myelitis and optic neuritis [20, 21].

## Management

### Immediate Management and Stabilization

#### Anatomical Reduction and Surgical Management

External immobilization is indicated for initial management of a suspected spine injury in the pre-hospital setting. After diagnosis of spine instability, rigid external cervical orthosis and spinal precautions (limiting movement) are used to prevent further injury. For infants with large heads, a backboard with occipital recess or thoracic padding is needed to prevent neck flexion while supine [5]. Patients with a fracture that is causing spinal cord compression from spinal canal narrowing may benefit from acute (< 24 h) or hyper-acute (< 12 h) reduction or surgical decompression as recent studies have demonstrated potentially improved functional outcomes [22••, 23].

In 2012, the multicenter, international, prospective cohort Surgical Timing in Acute Spinal Cord Injury Study (STASCIS) found surgical decompression within 24 h was safe and associated with improved American Spinal Injury Association Impairment Scale (AIS), a standardized tool for predicting ambulation after SCI, of at least 2 grades at 6 months after injury [24, 25]. Subsequent studies suggest that early surgery within 8 h may provide additional benefit [26, 27, 28••].

In the trial, surgical decompression was performed either through closed reduction with traction or open surgical reduction. Closed reduction is performed by cranial fixation with controlled distraction to re-align the spine, decompressing the spinal cord, and preventing continued injury. Pre-reduction MRI can evaluate for disc herniation that may require operative treatment. If a misaligned spine is not able to be reduced with traction, then operative reduction is indicated [25]. Results from the Spinal Cord Injury–Prospective Observational European Multicenter (SCI-POEM) study are expected to be published in late 2022 and will assess outcomes with hyper-acute surgical intervention (< 12 h) [29].

Cervical traction begins by application of a halo or tongs. Halo is preferred for children because it allows multiple skull pins to be applied at a lower pressure per pin, as opposed to the two-pin fixation of tongs. In addition, halo allows for fixation to a vest for long-term external fixation, if needed. For jumped or perched facets, a patient is placed in slight flexion with distraction. Increased weight is applied with serial radiographs until the distracted facet is realigned or the adjacent disc spaces appear over-distracted. Care must be used to not over-distract the spine, which may cause further injury. A disc could herniate during traction, causing acute worsening of injury. Frequent neurologic examinations during traction are critical. Once the spine is reduced, the patient may remain in traction or placed in a halo vest until surgery. Most dislocations occur because of significant disc or ligamentous disruption, which will not heal with time. Significant disc or ligamentous injury requires internal fixation and arthrodesis to achieve spine stability.

Most patients with spinal cord injury have spine fracture or disruption of disc or ligaments. Spine fracture without disc or ligament injury may heal with external immobilization for 6 to 12 weeks. External fixation devices for cervical fractures include halo vest and rigid external cervical orthosis. For fractures that span the cervical thoracic junction, a thoraco-lumbar sacral orthosis (TLSO) or cervical thoracic orthosis (CTO) is indicated.

Internal fixation may be indicated for unstable fracture or fractures requiring operative decompression. Internal fixation provides stability until solid arthrodesis between unstable vertebrae. Surgical treatment may be anterior, posterior, or both, depending on the extent of the injury, need for anterior decompression, and ability to reduce the fracture dislocation. Internal fixation may be augmented by external orthosis until the bone is healed. Guidelines exist for surgical indication to fuse traumatic injuries; however, the extent of fixation and arthrodesis is typically patient and surgeon dependent with goal to provide adequate fixation to prevent dislocation during the bone healing process [30, 31]. Bone healing is induced by compression and immobilization. Fracture segments that move or are not held in compression during the healing process may form a fibrous union called pseudoarthrosis that may not be stable long-term. Surgical options for rigid fixation include anterior vertebral body screws held to a buttress plate, anterior interbody cage to provide structural stability, and posterior pedicle screw, pars screw, or lateral mass screw fixation between vertebra connected by posterior rod construct.

Cervical vertebral fractures predict potential vertebral artery injury [32]. CT angiogram is often used to detect dissection or intraluminal thrombus. Treatment is directed towards prevention of progression of intraluminal dissection and prevention of thromboembolism, often using a single antiplatelet agent. A technique to increase perfusion of the spinal cord is to place a lumbar intrathecal drain to lower intrathecal pressure to augment perfusion of the spinal cord. This technique was pioneered by vascular surgeons when treating aortic aneurysms that could block spinal radicular arteries, decreasing perfusion to the spinal cord. Preclinical studies in pigs and clinical human studies show promising results that this technique may decrease the extent of injury to the spinal cord; however, the technique is still experimental and not standard of care at this time [33, 34]. As with post-traumatic brain injury monitoring, intraspinal pressure monitoring and spinal cord autoregulation are currently being studied in SCI and targeted perfusion therapy may be on the horizon [35–38].

#### Spinal Shock

Acutely after SCI, a transient phenomenon referred to as spinal shock can occur. It is characterized by sudden loss of spinal reflexes and muscle tone below the level of injury [4]. The underlying pathophysiological mechanism in spinal shock is poorly understood. The onset is immediately after or hours after injury and can be varied in presentation depending on the acuity of injury [4]. Spinal shock is considered to be multi-phasic with variable presentation and resolution [39]. The presentation also depends on the level of injury and severity (complete vs incomplete). While the sacral reflexes may be intact with high cervical lesions, it is important to not confuse the presentation with sacral sparing as over time these reflex arcs may be impacted [40]. Spinal shock is initially characterized by areflexia/hyporeflexia within the first 24 h. Within 1 to 3 days after the injury, initial return of reflexes can be seen [39]. Further return of reflexes is typically a gradual process and often begins with delayed plantar response and can be followed by the cutaneous reflexes (bulbocavernosus, anal, and cremaster) [40]. Over the next few weeks, deep tendon reflexes may return and can follow the sequence of ankle, Babinski sign (replacing delayed plantar reflex), and patellar [39, 40]. The final phase of recovery includes return of the bladder reflex typically with the emergence of spasticity/hyperreflexia in severe cases [39]. The pattern of reflex recovery appears to be cutaneous polysynaptic reflexes followed by monosynaptic reflexes [40].

### ICU Management

#### Respiratory Management

Failure to maintain a patent airway and breathe effectively is common following SCI. The diaphragm is controlled by C3–C5, and intercostal muscles may also be affected. Patients may have a weak cough and difficulty controlling secretions. Many also have concomitant brain injuries, which can impair the ability to protect the airway with loss of central control of breathing. Additional pulmonary injuries including pneumothorax and direct laryngeal, tracheal, or vocal cord injuries may be present. Trauma is a known trigger for the development of acute respiratory distress syndrome (ARDS) [41].

Adults and children with high cervical lesions at or above C5 are likely to require immediate intubation due to respiratory arrest and should be considered for early intubation [2••, 42]. Unlike adults, children are more likely to experience progression of symptoms over the first few days due to the acute inflammatory response and development of edema; therefore, early intubation is preferred [2••]. If patients are not immediately intubated, close monitoring of their respiratory status is needed with both pulse oximetry and end tidal CO_2_ monitoring. Noninvasive ventilation is often not optimal due to the inability to protect the airway and control secretions.

Best practice for securing the airway has not been well studied in pediatric patients. In adults, it has been suggested that awake fiberoptic intubation may be ideal if intubation is not emergent [43]. This is not an option for most children too young to cooperate; sedation and analgesia are required to ensure safety. Selection of medications for intubation should be carefully considered given the risk of neurogenic shock. Avoidance of medications that may exacerbate vasoplegia and hypotension is important. Adrenal insufficiency has been reported in this population and must be considered when intubating [44]. Manipulation of the airway may result in vagal response leading to bradycardia, which may be exacerbated by hypoxia [45, 46]. Atropine may be required. It is important to ensure adequate intravascular volume. It is suggested to have intravenous fluids with a push/pull set up or rapid infuser as well as vasoactive infusions at the bedside for hypotension. It is advisable to have the most experienced provider in airway management attempt intubation first. Care must be taken to avoid movement of the cervical spine with inline manual stabilization by a second provider to ensure that the primary provider can focus solely on management of the airway. A head tilt maneuver should not be used. Video laryngoscopy should be considered to minimize movement; however, it should also be recognized that in some settings, this has prolonged time to intubation [2••, 6, 47]. Risks and benefits must be weighed based on the patient’s immediate clinical situation and availability of equipment. Many patients will require long-term mechanical ventilation [48]. Optimal timing of tracheostomy is not known but may be necessary for some patients [49]. Early tracheostomy may have advantages of minimizing continuous sedative and analgesic infusions and may facilitates earlier mobilization and rehabilitation. Long-term, diaphragmatic pacing may be an option to minimize or eliminate the need for ventilation for select patients [50, 51].

#### Hemodynamic Management

Neurogenic shock, a distinct phenomenon from spinal shock, is common following SCI and is characterized by profound vasoplegia and hypotension and often bradycardia. Adult guidelines for blood pressure (BP) management recommend maintaining goal MAPs > 85–90 for 1 week following injury, which is associated with improved neurological outcomes [52–54]. There are no specific pediatric recommendations for BP control, though it is reasonable to target MAPs equivalent to adult goals for age. In older children, targeting MAPs 85–90 is likely appropriate. Recommendations suggest ensuring BP is above the 5th percentile for age; however, targeting higher blood pressures may help to avoid intermittent measurements below goal [2••]. Cardiac arrhythmias, cardiac arrest, and EKG abnormalities have been reported and close monitoring is imperative [55].

No agent has single proven to be superior in targeting BP. Agents with both alpha- and beta-adrenergic effects such as norepinephrine may be ideal to help with both peripheral vasoconstriction and bradycardia. One study in adult patients showed that norepinephrine was comparable to phenylephrine to maintain blood pressures but norepinephrine showed better spinal cord perfusion and oxygenation (Table 1) [2, 9, 45, 47, 89, 90].

**Table 1:**
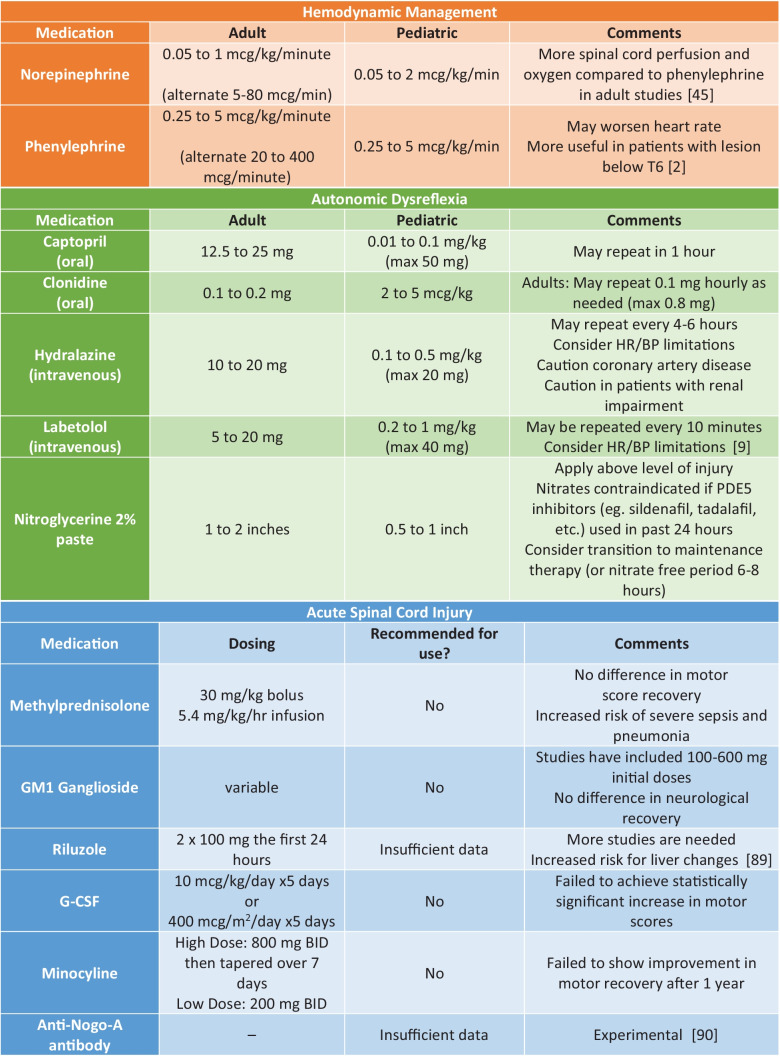
Selected pharmacological treatments for SCI

Future directions may be individualized BP goals based on optimal spinal perfusion pressures using lumbar catheters and pressure reactivity index (a surrogate measure of cerebrovascular autoregulation) [56]. Ongoing studies are evaluating optimization of spinal cord perfusion and oxygenation using infrared spectroscopy [57].

#### Pain

Managing pain can be very challenging in SCI. It is best to start with medications that do not impair respiratory drive such as acetaminophen and nonsteroidal anti-inflammatory medications, if the general clinical condition allows. For musculoskeletal pain, narcotics can be considered; however, caution must be used in patients without a definitive airway as they can cause respiratory depression and hypoventilation, which may be more profound if weakness is present. Medications targeting neuropathic pain should be used if there are characteristic features including burning, electric-like sensations, and allodynia [58]. Gabapentinoids, selective serotonin reuptake inhibitors, tricyclic antidepressants, and lidocaine patches can be considered [59]. Side effect profile must be considered when initiating these medications, including potential cardiac and behavioral complications. Partnering with a multi-disciplinary team experienced in pain management in this population is important.

#### Autonomic Dysreflexia

Autonomic dysreflexia (AD) is a life-threatening condition that can occur in individuals with SCI at the T6 level or above [60•]. With parasympathetic input cut-off, sympathetic activation is unopposed resulting in elevated systolic blood pressure with baroreflex-mediated bradycardia [61]. Patients present with sudden onset, severe headache with flushing and sweating above the level of injury and pale, cool skin with piloerection below the level of the lesion [62]. AD can lead to seizures, stroke, and intracerebral hemorrhage [63]. The most common cause is an overdistended bladder. A popular pneumonic to remember triggers is “6 Bs”: bladder (urinary tract infection, urinary retention, nephrolithiasis, blocked catheters), bowel (constipation, impaction), back passage (hemorrhoids and fissures), boils (skin damage), bones (fractures), and babies (pregnancy, sexual intercourse, breast feeding) [64]. The first step in managing AD is to eliminate noxious stimuli, focusing on known triggers. The patient should be positioned upright to promote an orthostatic drop in BP and any constrictors, such as tight clothing and dressing should be loosened [65]. If the patient remains hypertensive despite these measures, treat with fast-acting anti-hypertensives (Table 1). If the episode is lasting greater than 30 min, the patient should be moved to the ICU for aggressive management [66].

#### Bowel and Bladder

*Bowel Management* Depending on the level of injury, individuals will exhibit either upper or lower motor neuron bowel patterns. Upper motor neuron (UMN) injuries result in hyperreflexic bowels and spastic anal sphincters, leading to constipation and stool retention. Lower motor neuron (LMN) injuries result in areflexic bowels with an atonic external anal sphincter, leading to a mix of constipation and incontinence. Bowels do not always declare themselves immediately if the injury is in the low thoracic or lumbar regions. A bowel regimen should be started for regular daily timed bowel movements. An UMN program can include digital stimulation to trigger the retrocolic reflux to induce colonic emptying along with oral and rectal medications. Manual evacuation may be necessary for LMN programs [67].

*Bladder Management* Long-term bladder management varies depending on the level of injury. Individuals start in the ICU with a foley catheter to prevent excessive pressure in the bladder and kidneys and to allow for close monitoring of their volume status. Once an individual is stable, the patient should be transitioned to an intermittent catheterization program to protect from urinary tract infections and to facilitate normal stretch and contraction of the bladder [68].

#### Immobility

*Venous Thromboembolism (VTE) Prevention* As paralysis leads to significant venous stasis, the risk of VTE is elevated after SCI and further elevated with comorbid polytrauma with major fractures, traumatic brain injury, and the use of central venous catheters [69, 70]. The risk of VTE in pre-pubescent children is very low; thus, consensus is to treat them with mechanical prophylaxis. Chemoprophylaxis is used for children post-puberty and adults for 8 weeks, a time which is sufficient for the body to adapt to its new patterns [71].

*Pressure Wounds* Pressure wounds are common in SCI due to immobility, lack of sensation, and atrophy over bony prominences. The best prevention techniques are frequent repositioning and pressure relief measures. The standard of care is repositioning a supine individual every 2 h. More frequent repositioning or offloading is recommended when seated, at least every 30 min for a minimum of 30 s [72].

### Therapies Studied in Clinical Trials (Summarized in Table 1)

#### Steroids

Intravenous methylprednisolone (MPS) for neuroprotection and reduction of secondary injury remains controversial in tSCI. In prior studies, MPS initiated within hours of injury had been associated with short term motor score improvement; however, long-term benefits were not demonstrated after 6 or more months [73]. Subsequent structured analysis from NASCIS I and NASCIS II studies failed to demonstrate clinically significant improvement. Three randomized trials and one prospective observational study failed to show effect on motor function recovery at 6 weeks, 6 months, or 12 months [74–76].

Safety studies remain controversial. A Cochrane review of NASCIS II showed a potential increase in the risk for wound infection and gastrointestinal hemorrhage [76]. Subsequent analysis associates the use of high-dose steroids with harmful side effects including death [5, 12, 75]. NASCIS III compared 24-h versus 48-h MPS infusion trended towards a higher incidence of severe pneumonia in patients receiving 48-h infusion and a potential increase in severe sepsis, although this was not statistically significant [74, 77]. The impact on mortality remains unclear; however, increased risk for major complications in the MPS treatment group was noted [78]. Although steroids are not recommended in tSCI, they are used first-line for inflammatory etiologies.

#### GM1 Ganglioside

Gangliosides (GM-1) have been studied as a therapeutic option for SCI. Early animal studies demonstrated a positive response to therapy, with GM-1 therapy helping to enhance mitochondrial activity thought to protect against neuronal injury and degeneration. GM-1 is thought to be important for regrowth and regeneration of axons, which may help to repair and replace damaged tissue following SCI and may help restore function. An early 1990 study demonstrated improvement in motor score from baseline to 1-year follow-up [79]. However, a larger follow-up study failed to report a difference in proportion of patients achieving neurologic improvement [80]. Research for GM-1 therapy in neuroinflammatory disorders is still ongoing.

#### Riluzole

Recent studies suggest riluzole, a glutamatergic modulator, may be neuroprotective through sodium channel blockade and glutamate uptake modulation [30, 79, 81]. A systematic review identified three clinical studies evaluating potential for use in humans. Major findings included significant motor improvement with treatment but with associated risks, including elevated liver enzymes and bilirubin levels [82]. Unfortunately, a recent study terminated early due to recruitment issues and slow patient enrollment rates [54] (ClinicalTrials.gov: NCT01597518); however, analysis of the data collected is now underway.

#### Granulocyte Colony-stimulating Factor

Experimental animal studies hypothesize that granulocyte colony-stimulating factor (G-CSF) suppresses neuronal apoptosis [83]. Phase I/IIa studies compared two different doses to MPS. Intravenous administration minimizes risk of elevated white blood cell counts and potential splenic rupture when compared to subcutaneous administration. Patients receiving 10 mcg/kg/day doses observed higher American Spinal Injury Association Impairment Scale (AIS) motor scores; however, this finding was not statistically significant [82]. A recent phase III clinical trial was conducted to evaluate the effect of intravenous G-CSF 400 mcg/m^2^/day given daily for 5 days. A change in AIS motor score was not observed at 3, 6, or 12 months [82].

#### Minocycline

Preclinical animal studies of minocycline suggest neuroprotective effects. In murine models of SCI, minocycline is thought to be anti-inflammatory through reduction of caspase-3 activation, in turn reducing neuronal apoptosis and improving recovery early after SCI [84]. In human studies with SCI, minocycline administration at various timepoints within the first 24 h of injury did not affect functional recovery. A phase II clinical trial failed to show improvement in motor recovery after 1 year [82].

## Prognostication

**Fig. 1:**
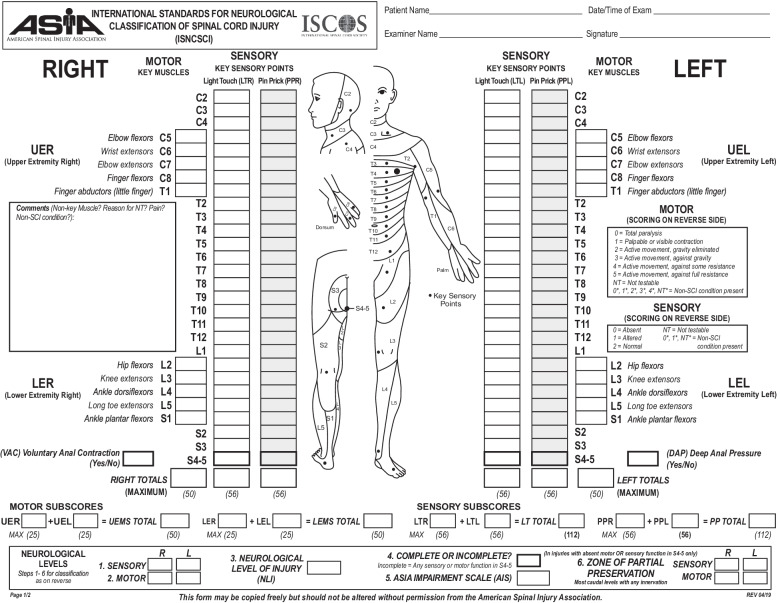
American Spinal Injury Association (ASIA) Form [84].

Early neurological examination is of utmost importance for prognostication with regard to ambulation, bowel/bladder function, and other self-care activities. The AIS classifies the preservation of motor function and sensation, with grade A being the most severe and D being the mildest (Fig. 1) [24, 85]. Preservation of sacral sensation indicates that an individual’s AIS grade may improve by one level. Individuals with lower AIS grades and those under 50 years old have significantly better chances of motor function restoration. MRI of the spinal cord can provide highly valuable information. The length of intramedullary hemorrhage, canal diameter at maximal spinal cord compression, and spinal cord swelling often correlate with the severity of impairment [86]. Another ancillary testing modality that may be useful includes neurophysiological studies such as somatosensory-evoked potentials (SSEPs) and transcranial magnetic stimulation (TMS). SSEPs assess the integrity of posterior column function, whereas TMS directly tests the motor pathways. The decision to use one or another depends on the clinical spinal syndrome.

*Early Rehabilitation* Rehabilitation plays a significant role in recovery after SCI, starting immediately after acute stabilization. Early upright mobilization should be initiated gradually using a tilt table. Orthostatic hypotension can be managed with abdominal binders, compression stockings, and premedication with drugs such as midodrine [87]. Respiratory therapists can begin to slowly minimize vent settings, while speech therapists work with individuals on breath control techniques. Physical and occupational therapists begin passive and active range of motion exercises and when appropriate strengthening exercises. Orthotic devices should be implemented immediately to allow for neutral positioning of limbs to prevent contracture formation. Although efficacy was demonstrated in a small case series of three individuals, epidural electrical stimulation in conjunction with aggressive targeted therapy was able to restore a trunk and leg motor function after complete sensorimotor paralysis in a select group of patients [88••]. Novel and innovative therapies such as these are on the horizon and provide hope for the future of SCI treatment.

## Conclusion

Although rare, SCI is a potentially devastating neurological emergency requiring rapid identification, assessment, and management. Given the potential of severe functional deficits or even death without proper care, management of SCI remains a subject of utmost importance in neurocritical care. Given the paucity of high-quality interventional studies in SCI, much of the management of SCI is driven by experience and expert consensus. Further research in early interventions, pathomechanistically targeted therapeutics, and acute rehabilitation is needed to facilitate development of robust evidence-based recommendations and guidelines.
